# Eosinophilic Granulomatosis with Polyangiitis in an 8-year-old Girl Manifesting as Hypereosinophilic Syndrome with Myocarditis, Stroke, and Subsequent Orbital Involvement

**DOI:** 10.15388/Amed.2023.30.1.5

**Published:** 2023-02-27

**Authors:** Aleksandra Panina, Elīna Ligere, Elīna Aleksejeva, Zane Davidsone, Elizabete Cebure, Irina Erdmane

**Affiliations:** University of Latvia, Faculty of Medicine, Riga, Latvia; Children’s Clinical University Hospital, Department of Paediatric Cardiology and Cardiac Surgery, Riga, Latvia; Riga Stradins University, Department of Paediatrics; Children’s Clinical University Hospital, Department of Paediatric Pulmonology and Allergology, Riga, Latvia; Riga Stradins University, Department of Paediatrics; Children’s Clinical University Hospital, Department of Paediatric Rheumatology, Riga, Latvia; 7Riga Stradins University, Department of Paediatrics; Children’s Clinical University Hospital, Department of Paediatric Haematology, Riga, Latvia; Riga East Clinical University Hospital

**Keywords:** paediatric hypereosinophilic syndrome, eosinophilic myocarditis, stroke, Churg–Strauss syndrome, orbital involvement

## Abstract

Hypereosinophilic syndrome (HES) is a heterogeneous group of disorders characterized by peripheral blood eosinophilia of 1.5 × 10^9^/L (1,500/μL) or greater, with evidence of end-organ damage attributable to eosinophilia (e.g., heart, liver or lung) with no other cause for the end-organ damage [1]. Eosinophilic granulomatosis with polyangiitis (EGPA) is a rare disorder that may affect multiple organ systems (lungs, heart, kidneys, or the nervous system). The disorder is characterized by hypereosinophilia in the blood and tissues, inflammation of blood vessels (vasculitis), and the development of inflammatory nodular lesions called granulomatosis [2]. We report a case with a 9-year-old girl presenting with severe hypereosinophilia, ischemic stroke, right-sided hemiparesis and myocarditis treated with methylprednisolone, enoxaparin, rivaroxaban and carvedilol. The patient recovered successfully from myocarditis and stroke but manifested with right-sided orbital involvement as pre- and post-septal orbital cellulitis 10 months later with necrotizing granulomatous perivascular chronic infiltration with eosinophilic infiltration treated with methylprednisolone and subsequent mepolizumab with successful remission of orbital involvement, but severe exogenous Cushing’s syndrome and myocardial fibrosis.

## Santrauka.

Hipereozinofilinis sindromas (HES) yra heterogeninė sveikatos sutrikimų grupė, kurią apibūdina periferinio kraujo eozinofilija 1,5 × 10^9^/L (1,500/μL) (arba dar didesnė vertė), kai yra duomenų apie neatitaisomą organų (pvz., širdies, kepenų ar plaučių) pažeidimą, kurį galima priskirti eozinofilijai, kai negalima išskirti jokios kitos šios neatitaisomos žalos priežasties [1]. Eozinofilinė granuliomatozė su poliangitu (EGPA) yra reta liga, galinti paveikti daugelio organų sistemas (plaučius, širdį, inkstus arba nervų sistemą). Šią ligą galima nustatyti iš hiperozinofilijos kraujyje ir audiniuose, kraujagyslių uždegimo (vaskulito) arba kai atsiranda uždegiminių mazginių darinių, vadinamų granuliomatoze [2]. Pristatome atvejį, kai 9 metų mergaitei pasireiškė sunki hipereozinofilija su išeminiu insultu, dešiniąja hemipareze ir insultu; gydoma buvo metilprednizolonu, enoksaparinu, rivaroksabanu ir karvediloliu. Pacientė sėkmingai pasveiko nuo miokardito ir insulto, tačiau jai po 10 mėnesių tebebuvo dešiniosios pusės orbitalinis sutrikimas (preseptalinis ir postseptalinis orbitalinis celiulitas), dėl kurio išsivystė nekrotizuojanti granuliomatinė perivaskulinė chroniška infiltracija, ji gydyta metilprednizolonu ir vėliau mepolizumabu. Orbitalinis uždegimas sėkmingai atslūgo, tačiau pasireiškė sunkus egzogeninis Kušingo sindromas ir miokardinė fibrozė.

## Introduction

*Hypereosinophilic syndrome* (HES) is a spectrum of myeloproliferative disorders, which is characterized by persistent and marked blood eosinophilia and damage to multiple organs due to eosinophilic infiltration. The criteria for HS consist of (1) persistent eosinophilia of >1500 eosinophils/mm^3^; (2) exclusion of other conditions causing eosinophilia; (3) signs and symptoms of organ system dysfunction (e.g., heart, liver, or lung). *Eosinophilic myocarditis* is a rare and potentially lethal disease characterized by eosinophil infiltration of the myocardium. Paediatric myocarditis remains challenging from the perspectives of diagnosis and management [ 3]. Clinical manifestations present a wide spectrum, ranging from mild symptomatology to severe symptoms such as retrosternal pain, rhythm disturbances, and sudden death [4]. Multiple etiologies exist, with most cases being related to viral illnesses [3]. Poor prognosis is usually associated with cardiac involvement and malignant transformation of blood cells. Eosinophilic granulomatosis with polyangiitis (EGPA) is a systemic small-to-medium-sized vasculitis associated with asthma and eosinophilia. Histologically, EGPA presents tissue eosinophilia, necrotizing vasculitis, and granulomatous inflammation with eosinophil tissue infiltration and commonly involves the upper airway and lung parenchyma, peripheral neuropathy, cardiac disorders, and skin lesions. Anti-neutrophil cytoplasmic antibodies (ANCA) are positive in 40% of cases [ 5]. The 2022 American College of Rheumatology/European Alliance of Associations for Rheumatology Classification Criteria for Eosinophilic Granulomatosis with Polyangiitis are a maximum eosinophil count ≥1×10^9^/L (+5), obstructive airway disease (+3), nasal polyps (+3), a cytoplasmic antineutrophil cytoplasmic antibody (ANCA) or anti-proteinase 3-ANCA positivity (−3), extravascular eosinophilic predominant inflammation (+2), mononeuritis multiplex/motor neuropathy not due to radiculopathy (+1) and haematuria (−1) [ 6]. Paediatric EGPA is rare. In the French cohort, the biggest paediatric EGPA series with 14 patients is described with a median age at diagnosis of 12.3 years, where the organ systems most frequently involved were the upper airway (85%), skin (71%), digestive tract (64%), and heart (57%), four of the fourteen children were positive for ANCA (30.7%). Four of the fourteen children were positive for ANCA (30.7%), 64% had a relapse of the disease and 1 patient died [ 7].

## Case report

An 8-year-old patient presented to the Emergency Department of the Children’s Clinical University Hospital (CCUH) in the late evening in August 2021, complaining of an acute febrile illness for 3 weeks with an intermittent febrile body temperature above 38 ˚C, accompanied by submandibular oedema, abdominal pain, loose stools, joint pain, and pain and tightness in her chest. The general practitioner had previously diagnosed an Epstein–Barr virus infection clinically, but the EBV IgM was negative, while the EBV IgG was positive. The patient was treated with amoxicillin/clavulanate for a week and has since then developed persistent diarrhoea. The patient had a diagnosis of bronchial asthma for 1 year with a need for the intermittent use of inhalation with salbutamol and fluticasone propionate due to a dry cough, but with no severe asthmatic episodes. On admission, her body temperature was 37.7˚C, tachycardia 165 beats/minute, normal arterial pressure 110/50 mmHg, no cardiac murmurs or pulmonary rales audible, a respiratory rate 40 times per minute, SpO2 96%, no hepatomegaly and negative meningeal symptoms. The patient’s blood tests showed HES, fulfilling the criteria: severe leucocytosis WBC 102.34 x10^3^ uL, eosinophilia 24.9%, absolute eosinophilia 25.53x10^3^/uL, thrombocytopenia 72x10^3^/ uL, erythrocytes 4.32x10^6^/ µL, CRP 87 mg/L, LDH 994 U/L, ferritin 1306 ng/mL, total IgE > 2000 U/mL, EGA 48 mm/h, pANCA and cANCA negative, ANA positive 1:1280, EBV IgM positive, troponin I 1880.4 pg/ml (acute myocardial involvement/myocarditis suspected) and ProBNP 6786 pg/ml. While at the emergency department, the child complained about generalized pain in her body and was not able to move her right hand or her right leg. *The computed tomography (CT) of the head* without contrast showed no signs of haemorrhages but thickened mucous membranes in all paranasal sinuses. *Magnetic resonance imaging (MRI)* of the head was performed and revealed: acute cerebral infarcts of various sizes, localized in the cerebellum, in all lobes of the large hemispheres in the anterior, posterior, and inferior border basins. The finding might be consistent with hypereosinophilia syndrome.

To exclude the diseases of the hematopoietic system, blood tests for flow cytometry and cytogenetics of peripheral blood were sent to the laboratory at Vilnius Hospital and excluded the myeloproliferative process. No significant findings related to HES were found by flow cytometry in the peripheral blood and bone marrow. No signs of clonality in TCRB, TCRG or TCRD genes were detected. In the bone marrow biopsy, hypercellularity was found with a predominance of eosinophils, but no atypical eosinophils, platelets or increased blast count were found, corresponding to secondary HES.

Serology of the blood was performed to exclude parasitic diseases (*toxoplasma, toxocara, taenia solium, trichinella or echinococcus*), and cryptococcus antigens in the blood were excluded. Other probable causes such as tuberculosis, *entero* and *coxacki*, and *HIV* viruses were excluded. The patient had positive Toxocara canis IgA and a subsequent positive Western blot test. She, therefore, also received treatment with albendazole to treat the possible cause of hypereosinophilic syndrome.

Echocardiography showed a normal cardiac anatomy with slightly decreased functioning of the right ventricle (TAPSE 14 mm), hypertrophy of the right ventricle (RV) (RV free wall 11 mm), normal functioning of the left ventricle, but mild hypertrophy of the ventricular septum.

Her cardiac MRI was consistent with a presentation of acute myocarditis: late gadolinium enhancement, subendocardially diffuse in the right ventricle, and also in the inferior and lateral wall of the left ventricle, ejection fraction (EF) of the left ventricle of 41%, hypokinesia in the apex and posterior wall of the left ventricle, hypertrophy of the free wall of the right ventricle and severe hypertrophy of the apex of the right ventricle, diffuse oedema of the right ventricle and local oedema in the anterior and posterior walls of the left ventricle.

Image 1.Cardiac MRI images

**Figure:**
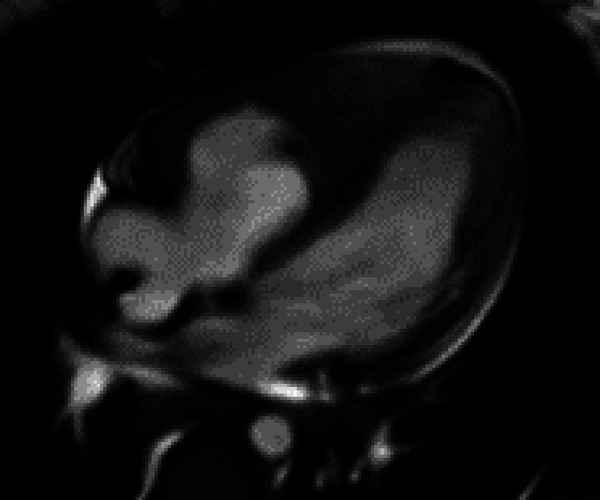

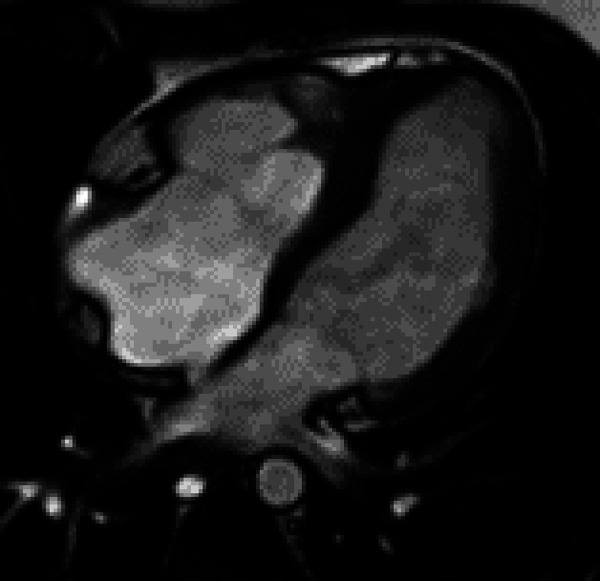
CMR 4-chamber cine images in diastole demonstrate improvement of the thickening of the right ventricular wall. August 2021 on the left and February 2022 on the right.

**Figure:**
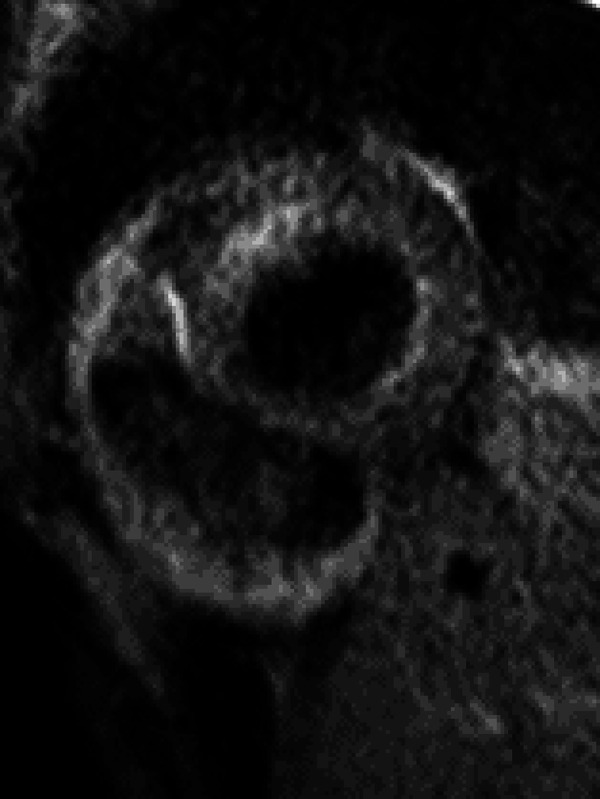

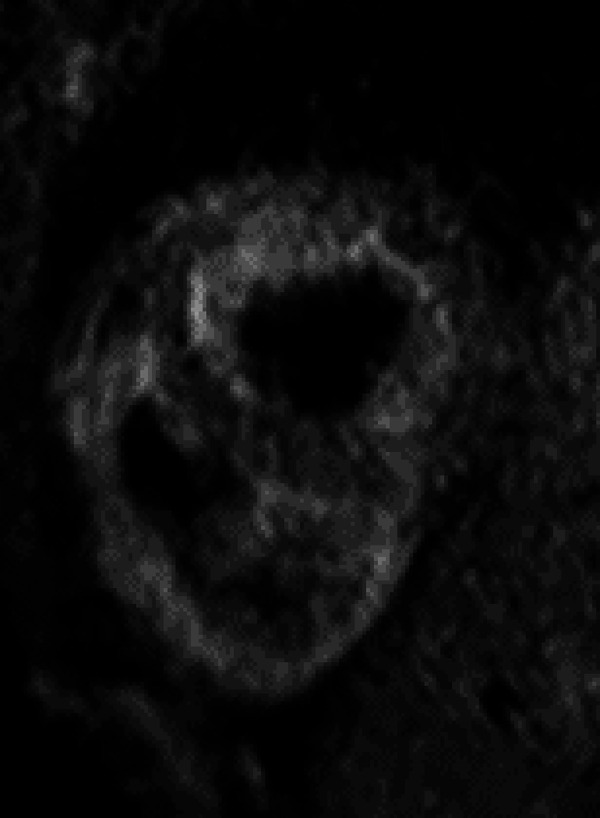
August 2021. T2 STIR demonstrating oedema of the RV wall and anterior wall of the LV. RV – right ventricle, LV – left ventricle.

**Figure:**
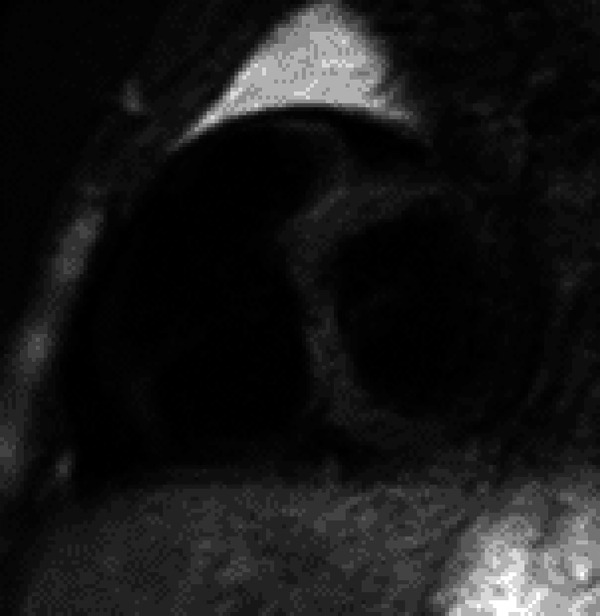

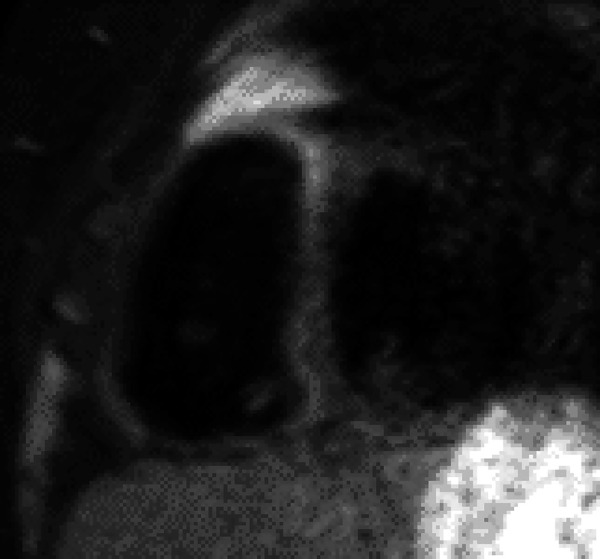
February 2022. T2 STIR demonstrating improved and nearly resolved oedema of the RV wall and anterior wall of the LV. RV – right ventricle, LV – left ventricle.

**Figure:**
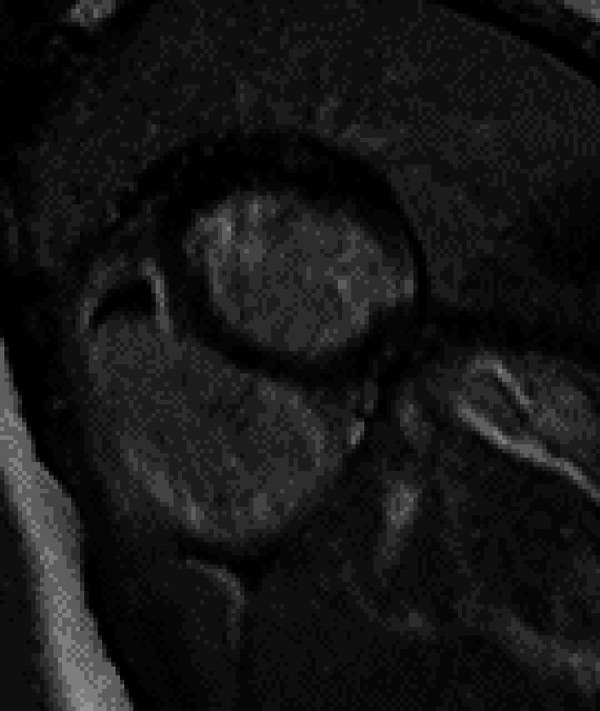

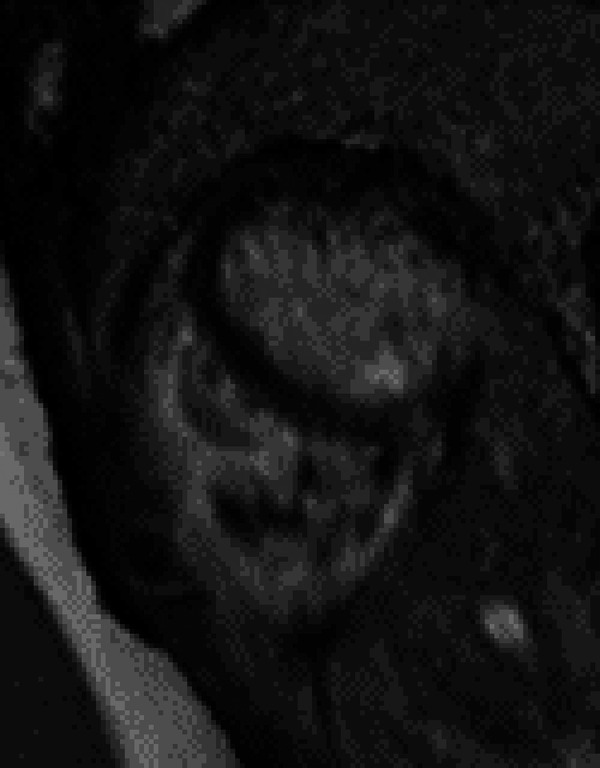

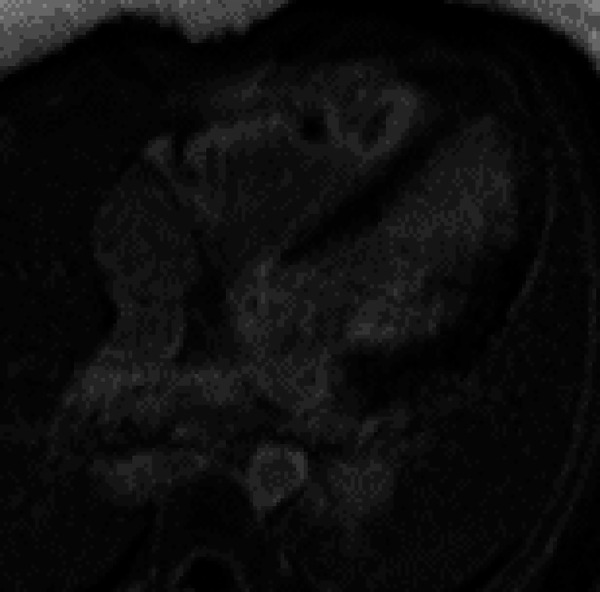

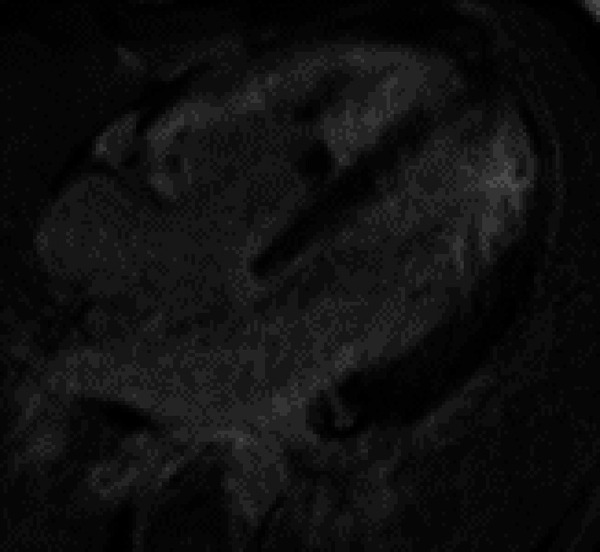
August 2021. Short axis and 4-chamber view. Late gadolinium enhancement (LGE) predominantly endocardial of the RV and LV wall. LGE of mitral valve papillary muscles and infero-lateral and apical wall of the LV. RV – right ventricle, LV – left ventricle.

**Figure:**
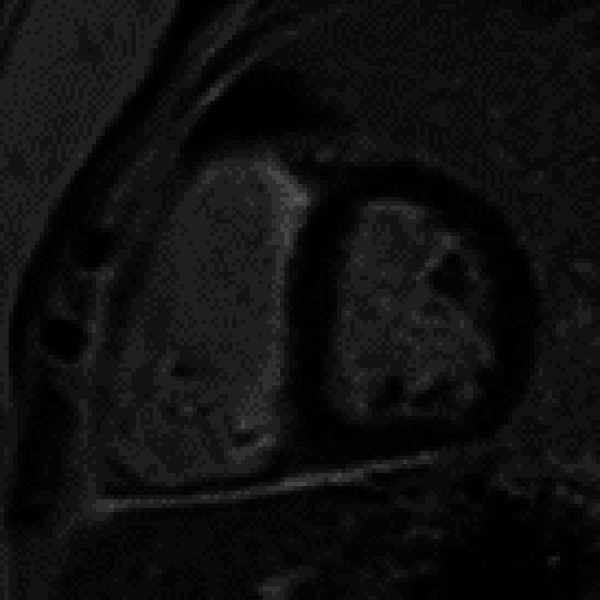

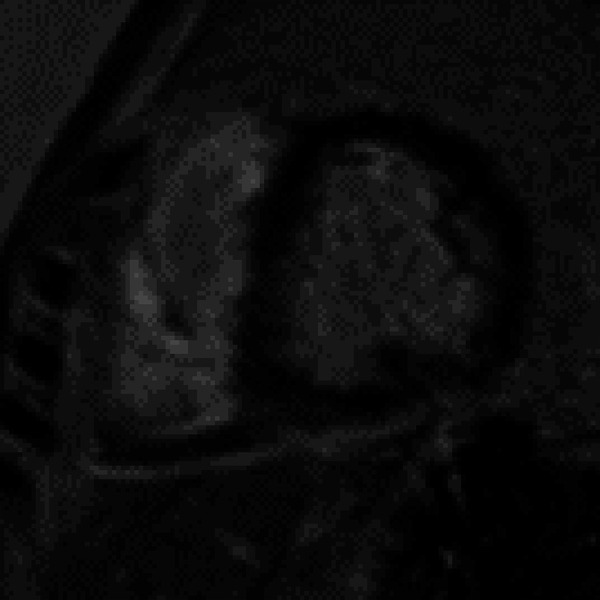

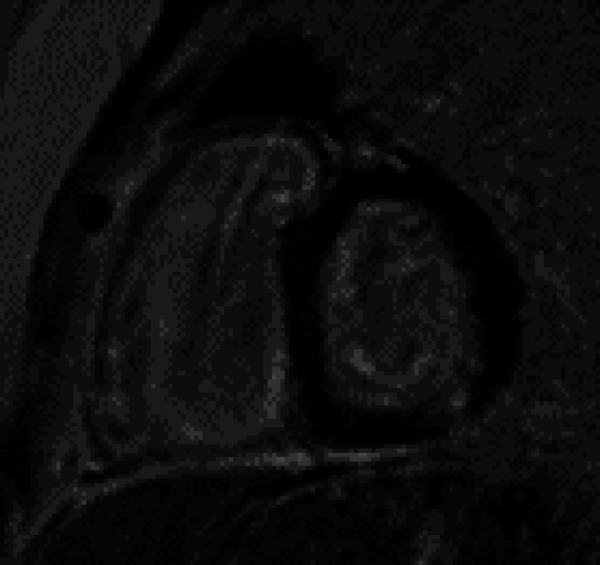

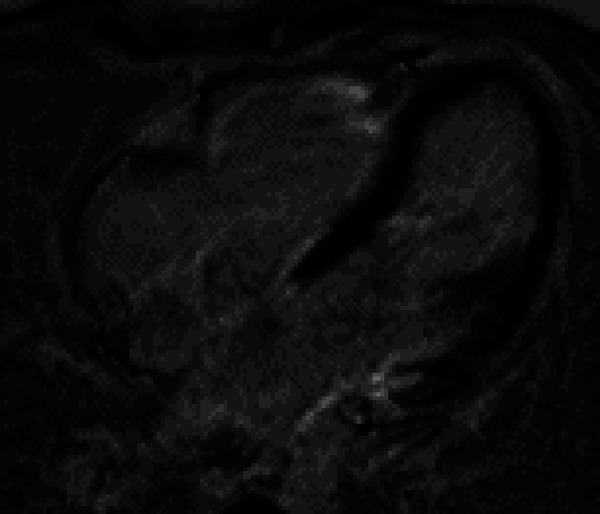
February 2022. Short axis and 4-chamber view. Images demonstrate improvement of late gadolinium enhancement (LGE) in the LV wall, however, to a lesser extent, but still present in the RV wall. LGE of mitral valve papillary muscles has resolved. LGE slightly decreased in the infero-lateral wall of the LV and resolved apically. RV – right ventricle, LV – left ventricle.

A thoracic CT showed right-sided pleural effusion up to 1.5 cm, small infiltrates in the right lower lobe segments S8 and S9. No evident hyperplasia of the intrathoracic lymph nodes.

Abdominal ultrasound and ophthalmological examinations were without pathology.

The patient’s treatment was discussed in a multidisciplinary consilium together with paediatric haematologists, paediatric rheumatologists, pneumonologists, cardiologists, neurologists, and immunologists. The diagnosis was hypereosinophilic syndrome with ischemic stroke and suspected eosinophilic myocarditis (the myocardial biopsy was not taken because of possible complications), but EGPA was considered as a possible differential diagnosis, as was the possibility of development of a myeloproliferative disorder.

Based on the “Guidelines for the Investigation and Treatment of Eosinophilia” [8]: the patient received methylprednisolone bolus intravenously followed by oral methylprednisolone 40 mg (14 days), then 36 mg (7 days) and 32 mg (7 days), with slow weaning then according to the scheme. Long term treatment with Medrol (slowly weaning during the next 3 months as suggested by the rheumatologist) led to the development of exogenous Cushing’s syndrome (initially the patient’s weight was 49 kg (+2.42z, BMI 25.4 kg/m^2^, but her weight increased afterwards to 74 kg (+3.24z, BMI 33.3 kg/m^2^). With this acute episode, the patient was treated in the CCUH for 41 days. She received a low molecular weight heparin (enoxaparin) therapeutic dose for 7 days, then a prophylactic dose which was changed to oral rivaroxaban for 6 months. Due to initial persistent tachycardia, myocardial hypertrophy, and inflammation, she received carvedilol 3.125 mg twice a day in the long term. The patient underwent rehabilitation and the paresis of the right leg already disappeared during the hospital stay, and after discharge, her right hand function improved fully within the next 3 months (the patient regained her right handwriting skills).

The patient’s progress was followed up by a cardiologist, haematologist, pneumonologist and neurologist. In the echocardiography, hypertrophy of the right ventricle had decreased. Two months after her discharge from the hospital, her blood count showed eosinophilia 0% and absolute numbers of eosinophilia 0x10^3^/uL.

The follow-up MRI of the heart performed on February 2022, 6 months after the first MRI, showed significant improvement in both ventricular functions, with left and right ventricular volumes within normal limits and without enlargement. Myocardial hypertrophy was still visualized in the right ventricle and was more pronounced in the distal segment of the apical region, where the residual abnormal myocardial gadolinium enhancement with a subendocardial oedema had markedly decreased but was still persistent. The myocardial morphological lesion had markedly regressed.

At the end of May 2022, the patient developed an oedema of the right upper eye lid. Tobradex (eye drops containing tobramycin and dexamethasone) were prescribed, but the oedema spread over the right side of the face and especially the right orbita during the next five days. The patient was hospitalized at the CCUH. Blood analyses showed eosinophilia 16.7%, leukocytes 10.74 × 10^3^, erythrocytes 5×10^6^ µ/L, thrombocytes 460 × 10^3^ µ/L and CRP 1.61 mg/L. The ophthalmologist noted that the right eye was sensitive to the left and left downward view, and that there was restriction of movement to the upward view, but the left eye was mobile to a lesser extent. The left eye had a full range of movement. A conclusion of right sided pre-septal and post-septal cellulitis was made. The otolaryngologist detected that the mucous membrane was pink and slightly oedematous.

To clarify the inpatient diagnosis, the patient underwent a biopsy of the inflammatory foci: in the ethmoid tissue there was constant lymphohistiocytic and eosinophilic inflammation with 30–50% eosinophilic ipsilateral infiltrate cells; granulomatous and chronic perivascular inflammation with eosinophils was found in the striated muscle tissue, which might be consistent with EGPA. Based on scientific articles, the early use of steroids (this patient received Medrol therapy) for a brief period could help to shorten hospitalization and prevent the progress of the inflammation [9]. As the patient had Cushing’s syndrome, and according to the bone marrow and blood tests, the consensus was to continue treatment with prednisolone (Medrol) orally 60 mg/day and, in parallel, to initiate pathogenetic therapy with anti-Il-5 antibody mepolizumab subcutaneously due to the relapsing course of the disease, long-lasting corticosteroid side effects and the suitability of the drug for idiopathic HES, L-HES phenotype and HES-eosinophilic granulomatosis with polyangiitis overlap.

**Image 2.**
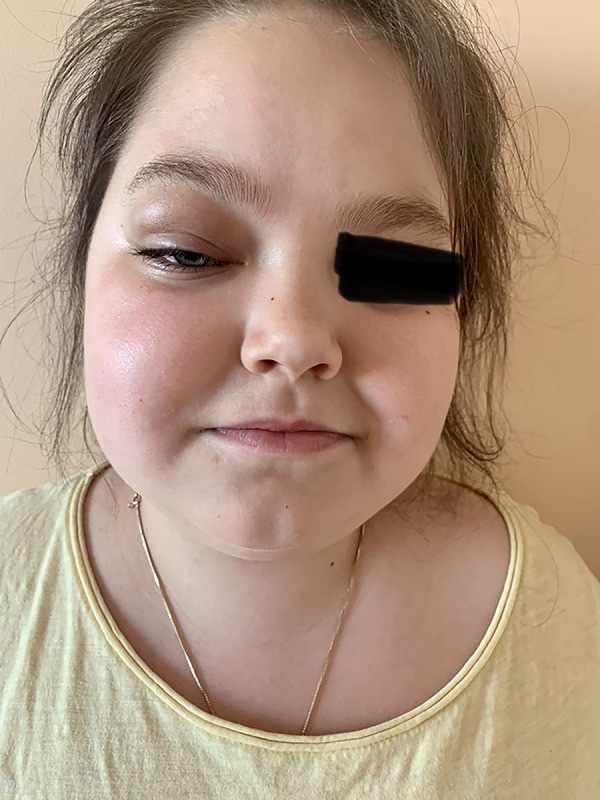

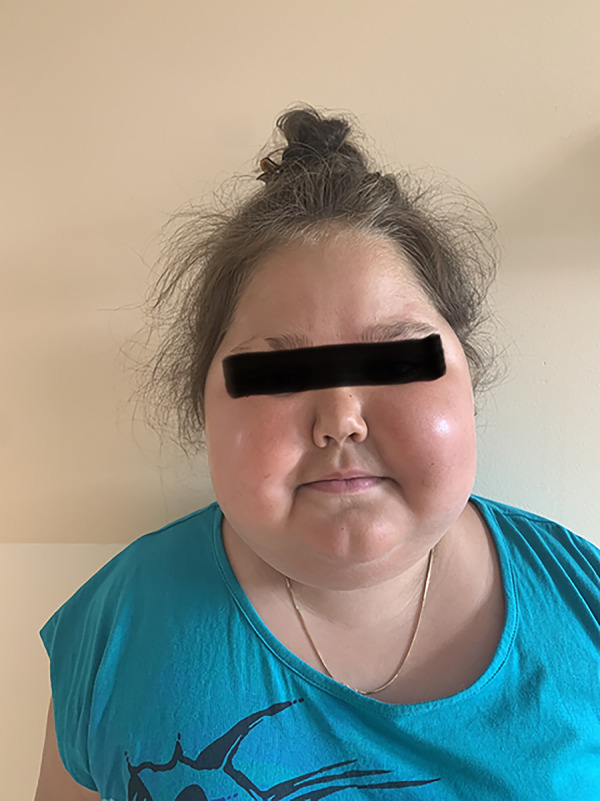
In this image one can see the patient before and after the orbital cellulitis.

## Discussion

Paediatric **e**osinophilia is associated with a variety of conditions: allergic, infectious, and neoplastic disorders with a wide diagnostic differential and limited data on hypereosinophilia in paediatric patients. EGPA is a type of ANCA-associated small-to-medium-sized vasculitis associated with asthma and eosinophilia. The most common manifestations in children are seen in the respiratory tract (69%), cutaneous tissue (61–62%), the gastrointestinal tract (46%), the cardiovascular system (46%), in paranasal sinus abnormality (38%), arthritis/arthralgia (38%), and the peripheral nerves (15%) [7,10,12]. Histologically, EGPA presents tissue eosinophilia, necrotizing vasculitis, and granulomatous inflammation with eosinophil tissue infiltration and commonly involves the upper airway and lung parenchyma, peripheral neuropathy, cardiac disorders, and skin lesions. Anti-neutrophil cytoplasmic antibodies (ANCA) are positive in only up to 40% of paediatric cases [5,7,10,11]. The pattern of organ involvement and clinical outcomes of patients with EGPA can differ depending on their ANCA status, which may reflect the different pathogenic mechanisms underlying ANCA-positive *vs* ANCA-negative EGPA [12]. The pathogenesis of the paediatric hypereosinophilic syndrome and EGPA is multifactorial. The disease can be triggered by exposure to a variety of allergens and drugs with the possibility of a genetic background and can lead to upregulation of different interleukins such as IL-4,IL-13 and IL-5. EGPA has a good response to glucocorticoids. Newer treatment options include anti-IL-5 antibodies (mepolizumab) and anti-CD-20, a B cell-depleting agent (rituximab), which have been reported in several case series [13].

Central nervous system (CNS) involvement in paediatric EGPA is rare with the main neurological manifestations being ischemic cerebrovascular lesions in 46 (52%) of the 88 described adult EGPA cases. Whereas among the 81 patients with assessable neurological responses, 43% had complete responses without sequalae [14]. Ocular manifestations in paediatric EGPA are rare and have been divided into two categories: ischemic vasculitis and orbital inflammatory pseudotumor. A diagnosis of orbital involvement secondary to CSS can be confirmed with the biopsy of an extraocular muscle, lacrimal gland, or orbital mass. The key to identifying EGPA in histology is necrotizing vasculitis with an extravascular infiltration of eosinophils. The presence of systemic findings can also help to determine the cause. CT and MRI results are less specific and demonstrate an enlargement of the retro-orbital space with swelling of the lacrimal glands and extraocular muscles. Cases in the literature have shown an excellent response to oral prednisone with almost complete resolution of orbital inflammation [15].

The prevalence and incidence of HES is unknown, but it is considered a rare disease in children and affected adults between 20 and 50 years old, with a 9:1 male-female ratio [12] and EGPA is a rare systemic vasculitis in children [16]. Statistical data and scientific publications show that HES and EGPA are exceedingly rare. As can be seen, EGPA can mimic hypereosinophilic syndrome and it is difficult to come to a true diagnosis from laboratorial data or other informative measurements and difficult to differentiate without histology and a negative ANCA. In our clinical case, only after orbital involvement and the biopsy results could we understand that the patient had EGPA, because HES and EGPA might have similar stages. When analyzing the scientific papers on the effects of hypereosinophilic syndrome on different organ systems, one can see that the involvement of the lung and heart or lung and nervous system are more frequently described, but the involvement of the 3 consecutive systems is rare, and a stroke due to this syndrome is the rarity. There are no specific biomarkers for the diagnostics of an EGPA jet. Grayson PC et al. conducted a study of biomarkers to evaluate disease activity while predicting relapse in patients with EGPA (eosinophil count, serum total IgE, ESR, and CRP). The study showed these markers have limitations and there is a need for new disease biomarkers [17]. Recommendations on the management, treatment, and prognosis in children are limited due to the rarity of hypereosinophilic syndrome and EGPA in children.

## Conclusions

Paediatric eosinophilic granulomatosis with polyangiitis (EGPA) and hypereosinophilic syndrome are multisystemic disorders with the possibility of overlapping and a wide range of life-threatening manifestations and a broad range of differential diagnostics with a need for multidisciplinary collaboration, urgent diagnostics and treatment and long term follow up to avoid a poor outcome. Mepolizumab might be safe and effective for controlling disease activity in relapsing EGPA and reducing and weaning off corticosteroids.
